# Why‐not‐doing‐high‐tech‐test Syndrome

**DOI:** 10.1002/jgf2.396

**Published:** 2020-11-03

**Authors:** Junki Mizumoto, Taro Shimizu

**Affiliations:** ^1^ Ehime Seikyo Hospital Ehime Japan; ^2^ Dokkyo Medical University Tochigi Japan

## Abstract

A 69‐year‐old man presented with fever, chill, and malaise. A thorough physical examination brought the correct diagnosis of psoas abscess to light. In this case, a physical examination is the only way to correct diagnosis.

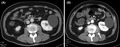

## 1 INTRODUCTION

A 69‐year‐old Japanese man presented with fever, shaking chill, and malaise. He was well until 3 days before presentation, when he developed gradually worthening general malaise. In the morning of the presentation day, the patient developed fever and dull pain over the entire chest, abdomen, and back, which disabled him to walk by himself. Because of the worsening symptoms, the patient presented to the emergency room (ER). On questioning, he denied palpitation, headache, diarrhea, constipation, melena, hematochezia, cough, and sputum.

Fever is undoubtedly one of the most common symptoms for primary care physicians. Fever itself is a low‐yield finding, or can produce an enormous variety of differential diagnoses. Thus, accompanying symptoms are of great importance in seeing febrile patients. For example, dyspnea, cough, and chest pain are usually related to respiratory diseases such as pneumonia and pulmonary embolism. No existence of organ‐focused problems also conveys significant information. Representative examples of febrile diseases without localized symptoms include miscellaneous viral infection, afferent infectious diseases, bloodstream infection, vasculitis, hematological malignancy, functional hyperthermia, and iatrogenic fever. In this case, aches and pains throughout coincided with fever. Assessment of the whole‐body pain should begin with precise identification of anatomical construction which is suffering. What clinicians should differentiate includes myalgia, ostalgia, arthralgia (intraarticular or extraarticular pain), neuralgia, angialgia, general malaise, and mid‐trunk pain due to aorta.

Attentive clinicians should focus on the presence of the patient shaking badly. Shaking chill is a very important indicator of bacteremia. All patients with shaking chill should be suspected to develop severe bacterial infection, so as not to miss an appropriate period of treating this critical condition.

His past medical history included myocardial infarction 12 years before, stroke 9 years before, and uncontrolled type 2 diabetes mellitus with unknown time of onset. He stopped visiting any healthcare services for 2 years because of his financial issues. He did not take any regular medication. He had no known drug and food allergy. He smoked 40 cigarettes daily since he was seventeen, but he quitted smoking when he was 60 years old. He seldom drank alcohol.

The patient is a cellular immunocompromised host given his diabetes mellitus uncontrolled for a long time. Hence, he has a high risk of severe bacterial infection, which includes necrotizing fasciitis with or without osteomyelitis, bloodborne infection and abscess formation, and emphysematous infection with cystitis, pyelonephritis, or cholecystitis. His past heavy smoking history increases the likelihood of a respiratory infection such as pneumonia and lung abscess.

He also has a high risk of cerebrovascular and cardiovascular events. Aortic dissection sometimes produces fever, which may be the only manifestation of that disease. An acute phase of ischemic stroke and hemorrhage of the central nervous system may present with fever. However, if brain stroke had happened, neurological focal symptoms may have been present.

Finally, the patient admitted that he had no medication nor alcohol. Thus, drug‐induced fever or withdrawal from alcohol or a certain drug such as benzodiazepine and antipsychotics is less likely, although we should not easily rule out even if the history from the patient seems reliable.

On brief physical examination, the patient looked diaphoretic and mildly lethargic with Glasgow Coma Scale of 14 (E3, V5, M6). His body temperature was 39.0°C, blood pressure 110/70 mm Hg, pulse 90 beats per minute and regular, respiratory rates 35 per minute, and oxygen saturation 93% while breathing ambient air. Lungs were clear to auscultation bilaterally. Cardiovascular examinations revealed normal heart sounds. The abdomen was flat and soft. There was no abdominal tenderness.

**Figure 1:**
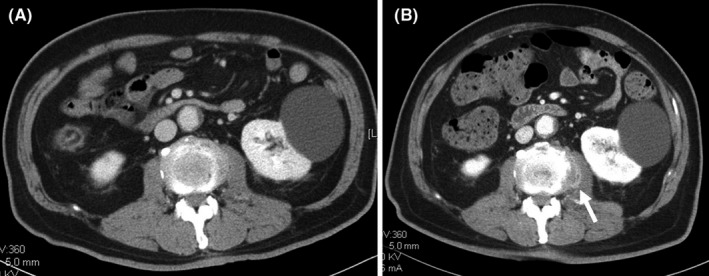
A, A contrast CT scan on admission. Abscess was not detected. B, A contrast CT scan on day 15. Psoas abscess was seen (white arrow)

Laboratory data (Table 1) revealed the white blood cell count of 15.2 × 10^3^/µL (normal range, 3.0‐8.5 × 10^3^/µL), the platelet count of 10.5 × 10^4^/µL (normal range, 15.0‐36.1 × 10^4^/µL), serum lactate dehydrogenase level of 341 IU/L (normal range, 106‐211 IU/L), serum gamma‐glutamyl transpeptidase level of 82 IU/L (normal range, 4.7‐52 IU/L), serum blood glucose level of 286 mg/dL (normal range, 74‐106 mg/dL), plasma brain natriuretic peptide level of 148.5 pg/mL (normal range, −18.4 pg/mL), and serum C‐reactive protein level of 6.37 mg/dL (normal range, −0.3 mg/dL). Alanine aminotransferase level, creatinine kinase level, creatinine level, and albumin level were within normal ranges. Arterial blood gas analysis data revealed mild respiratory alkalosis and corrected lactate level of 3.2 mEq/L (normal range, 0.6‐1.4 mEq/L). On urine analysis, neither pyuria nor bacteriuria was not seen. The rapid influenza diagnostic test revealed a negative finding. The electrocardiogram was within normal limits. A chest and abdominal computed tomography (CT) scan with contrast enhancement showed no abnormal findings which might cause fever (Figure 1A). Transthoracic echocardiography revealed no abnormal findings, including vegetation. Lumbar puncture was performed without any complication, and spinal fluid with no abnormal findings was taken.

**Table 1:** Laboratory data on admission

		Normal range
WBC	15.2 × 10^3^/µL	3.0–8.5 × 10^3^
Hb	14.3 g/dL	10.8 to 14.9
PLT	10.5 × 10^4^/µL	15.0–36.1 × 10^4^
AST	39 IU/L	8 to 38
ALT	25 IU/L	4 to 44
LDH	341 IU/L	106 to 211
ALP	196 IU/L	104 to 338
ɤ‐GTP	82 IU/L	4.7 to 52
T‐Bil	1.5 mg/dL	0.2 to 1.2
Cre	0.85 mg/dL	0.4 to 0.8
BUN	17.4 mg/dL	7.0 to 18.0
TP	7.3 g/dL	6.5 to 8.2
Alb	3.7 g/dL	3.8 to 5.3
CPK	79 IU/L	29 to 192
Na	134 mEq/L	136 to 145
K	3.5 mEq/L	3.5 to 5.1
Cl	101 mEq/L	98 to 107
Glucose	286 mg/dL	74 to 106
CRP	6.37 mg/dL	0 to 0.30
BNP	148.5 pg/mL	0 to 18.4
PT‐INR	1.23	0.80 to 1.20
APTT	26.2 s	28 to 38
Arterial blood gas analysis data (breathing ambient air)		
pH	7.492	7.36 to 7.44
PaCO_2_	30.1 Torr	36 to 44
PaO_2_	66.9 Torr	80 to 100
cLac	3.2 mEq/L	0.6 to 1.4
HCO_3_	22.8 mEq/L	22 to 26
Base EXCESS	0.8 mEq/L	−2.5 to 2.5
Anion Gap	13.0 mEq/L	10 to 14
Urine analysis		
Specific gravity	1.010	
pH	7.0	
Protein	2+	
Glucose	4+	
Ketone	±	
Occult blood	±	
Bilirubin	−	
Leucocyte	−	
Nitrate	−	

Abbreviations: Alb, albumin; ALP, alkaline phosphatase; ALT, alanine aminotransferase; APTT, activated partial thromboplastin time; AST, aspartate aminotransferase; BNP, brain natriuretic peptide; BUN, blood urea nitrogen; cLac, corrected lactic acid; CPK, creatine phosphokinase; Cre, creatinine; CRP, C‐reactive protein; ɤ‐GTP, ɤ‐ glutamyl transpeptidase; Hb, hemoglobin; LDH, lactate dehydrogenase; PLT, platelet; PT‐INR, prothrombin time‐international normalized ratio; T‐Bil, total bilirubin; TP, total protein; WBC, white blood cell.

The patient's quick SOFA score was two, which indicated that the probability of his developing sepsis was increased. The elevated lactate level also supported this diagnosis. If the patient had hypertension on a daily basis, which often coincides with diabetes mellitus and other atherosclerotic risk factors, his blood pressure in the ER was relatively low, and distributive or hypovolemic shock should be considered.

Tachypnea indicates some possible underlying conditions: respiratory problems, acid‐base disturbance, pain, and so on. Clear lung sounds do not rule out pneumonia or other intrathoracic infection, especially when a patient has tachypnea and hypoxemia. Gram‐negative bacterial infection may be the combinations of fever and respiratory alkalosis because of endotoxin. Metabolic acidosis, including diabetic ketoacidosis, may present Kussmaul breathing, or rapid, deep, and labored breathing. This condition, however, was practically ruled out in this case because of the result of blood gas analysis.

While contrast CT is sometimes useful in search of the origin of infection, it is never a versatile diagnostic tool, especially when the clear clinical aim to perform this test is not intended. In this case, a detailed history and a thorough physical examination should be needed. All diagnostic tests without sufficient pretest probability have the potential of only producing noise information and enticing clinicians to fruitless “abnormal findings.”

Although transthoracic echocardiography is useful in understanding the patient's circulatory dynamics, it is of limited information revealing vegetation indicating endocarditis because the sensitivity is not so high. Infective endocarditis itself should be considered as a differential diagnosis when seeing febrile and hemodynamically unstable patient. It causes various types of circulatory disturbance, including fulminant pulmonary edema and decreased cardiac output due to acute aortic or mitral valve regurgitation, hypovolemia induced by fever and deteriorating general condition, and septic shock probably due to secondary infectious sources. A report from tertiary centers showed that 5.8% of patients with infective endocarditis developed septic shock at admission and 17.4% at any time during the clinical courses.^1^

Based on his symptoms and a long history of uncontrolled diabetes mellitus, he was tentatively diagnosed as bacterial infection with unrevealed focus. Two sets of blood cultures were taken, and fluid repletion and an intravenous administration of 2 g ceftriaxone every 12 hours were initiated. He was admitted for further evaluation and treatment.

Administration of antibiotics after blood culture is a reasonable choice because the patient is likely to develop sepsis. He had some risk factors of developing a severe bacterial infection. It was still unrevealed where the origin of the patient's fever lay, so close observation and repeated bedside history‐taking and physical examination should be planned.

On the second day in the hospital, a thorough bedside physical examination was performed. The patient lay supine with his left hip somewhat flexed. He complained of moderate back pain. His conjunctiva was pallor and his sclera was nonicteric. There were no petechiae on his conjunctiva, palate, buccal mucosa, tongue, and tips of fingers and toes. His pharynx and palatine tonsils were normal, and he had no trismus. There was no tenderness nor swelling in his thyroid and no lymphadenopathy was seen. Lungs were clear to auscultation bilaterally, without percussion dullness, nor other abnormal findings. Heart sounds were normal. There was no bruit over the neck, abdomen, and bilateral inguinal areas. His abdomen was flat and soft. There was no percussion tenderness, liver tenderness, nor hepatosplenomegaly. A joint examination of the extremities and digital examination showed normal findings. There was no skin rash. A digit examination revealed smooth and nontender prostate.

Percussion tenderness was noted over the second lumbar vertebrae with adjacent muscles. Left psoas sign and left obturator sign were both positive. Bimanual palpitation of his left kidney revealed mild pain while left costovertebral angle tenderness was not observed. Based on the physical examination findings above, he was suspected of psoas abscess and pyogenic spondylitis as the sources of his fever, regardless of negative findings of contrast CT scan.

This case seems to illustrate the power of physical examination. An inspection of the whole body conveyed important information. The patient's characteristic limb position was very suggestive of any abnormalities in the hip joint or the flexor muscles of the hip. This finding motivated clinicians to examine the hip joint in more detail. Psoas sign, or pain provocation by hyperextension of the hip, and obturator sign, or pain provocation by inner rotation of the hip, revealed the possibilities of inflammation of psoas muscle, internal obturator muscle, external obturator muscle, and adjacent intraperitoneal and retroperitoneal organs (eg, appendix, sigmoid colon, kidney, ovary, uterus, abdominal aorta, and its branch). Soft and flat abdomen decreased the likeliness of inflammation of intraperitoneal organs. Considering these findings and tenderness of vertebrae and adjacent muscle, a coincidence of psoas inflammation and vertebral inflammation is most likely.

Kidney infection, such as pyelonephritis and kidney abscess, may or may not develop back pain and sometimes present only subtle findings such as mild discomfort by bimanual palpitation. Although pyuria and bacteriuria are seen in most cases of kidney infection, some patients do not show abnormal urine findings. Kidney infection without pyuria and bacteriuria includes preceding administration of antibiotics, infection of parenchymas such as kidney abscess and focal bacterial nephritis, urinary tract obstruction due to renal calculus, and other causes, and sample collection error.

Both psoas abscess and pyogenic spondylitis are often unrevealed by an imaging test, especially in their early stages. Thus, it is not surprising that contrast CT on admission did not show any abnormality. Imaging test sometimes turns out to be positive one to two weeks after the development of the first symptom. In this case, continuation of antibiotics administration and close watching are recommended at this point.

**Figure 2:**
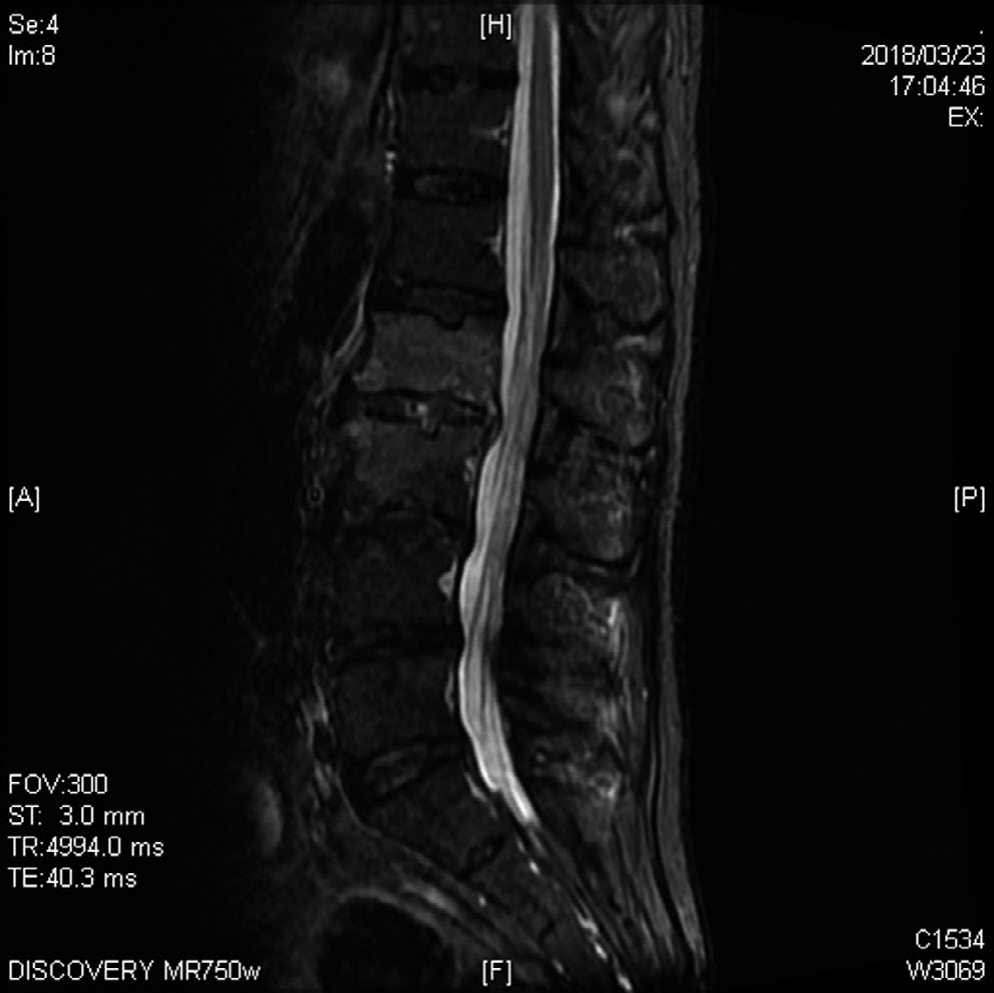
An MRI imaging of lumbar vertebrae on day 17. High intensity on STIR images was seen on the first and second vertebrae

The result of the blood culture of the patient turned to be positive for ceftriaxone‐sensitive *Klebsiella pneumoniae* in all the four bottles. Administration of ceftriaxone was continued. Other than low back pain, his condition seemed to be relatively well after the initiation of the antibiotics. However, on the day 13, he developed intermittent chill and rigor. Recurrent blood culture turned out to be positive for extended‐spectrum beta‐lactamase‐producing *Klebsiella pneumoniae*, and methicillin‐resistant *Staphylococcus aureus*. Antibiotics were changed to 1 g of meropenem every 8 hours and 1.25 g of vancomycin every 12 hours. On the day 15, an abdominal CT scan with contrast enhancement was reperformed, and abscess in the left psoas muscle was detected (Figure 1B). On the day 17, magnetic resonance imaging (MRI) scan without contrast enhancement revealed low‐intensity area on T1‐weighted images and high intensity on short T1 inversion recovery (STIR) images (Figure 2) on the first and second lumbar vertebrae.

Finally, the definitive diagnosis was made. Detection of polymicrobial infection including Gram‐negative rods indicates that abscess was formed secondarily due to the urinary or intestinal tract. Only intravenous administration of antibiotics showed limited treatment effect, and surgical intervention must be performed.

On the day 18, CT‐guided abscess drainage was performed. Bacterial culture of drained abscess turned out to be positive for *Klebsiella pneumoniae*. After the procedure, his fever was alleviated and his general condition improved. The drainage catheter was discontinued on the day 33. However, on day 35 in the hospital, fever recurred. A contrast CT scan on the day 37 revealed abscess stretching along the pathway of the drainage catheter. On day 42, surgical intervertebral disc drainage was performed. Fever did not recur after the surgery. He was discharged from the hospital on the day 106.

## 2 DISCUSSION

Psoas abscess is a relatively rare condition. Its incidence was reported as 4/1 000 000 in 1970s to 80s.^2^ Development of diagnostic tools including CT scan is thought to have raised the incidence, but the conditions are still not common.^3^, ^4^ The mortality rate of psoas abscess is as high as 8.1% to 8.7%.^4^, ^5^ Diagnostic delay may lead to septic shock and death, and an appropriate therapy must be initiated immediately.^6^ Diabetes mellitus is a risk factor of developing psoas abscess. 18.5% of patients with psoas abscess have diabetes mellitus.^3^

Psoas abscess is classified into two categories according to the pathogenesis: primary and secondary. Primary abscess results from systemic infection via hematogenous spread of a causative organism. Secondary abscess occurs due to direct invasion of the organism into adjacent organs, frequently including vertebrae, hip joint, gastrointestinal tract, and genitourinary tract.^6^ In fact, concurrent infection of psoas abscess and contagious vertebral osteomyelitis accounts for 35.5% of the total cases of psoas abscess.^3^

The clinical triad of psoas abscess includes fever, hip flexion contracture, and back, flank, or abdominal pain. Many patients with septic shock, however, do not satisfy the triad. In addition, elderly male patients tend to lack typical findings.^7^

Causative organisms of psoas abscess differ depending on its pathogenesis. In primary abscess, 71.4% of the cases are caused by Gram‐positive cocci. Otherwise, in secondary abscess due to urinary tract infection, 61.5% of cases are caused by Gram‐negative bacilli. 21.5% of total cases are polymicrobial infection, as is this case, and infection from gastrointestinal tract has the majority.^3^

Imaging tests such as CT and MRI are crucial to the diagnosis of psoas abscess. However, an early‐stage psoas abscess may be undetectable. The sensitivity of plain CT, contrast CT, and plain MRI from one to five days after the onset of symptoms is 33%, 50%, and 50%, respectively, although the sensitivity of each modality over 6 days is 100%.^8^ It suggests that negative findings of the contrast CT at presentation cannot rule out psoas abscess, and a careful observation and repeated imaging tests are needed.

A focused and speedy physical examination is definitely important for making the prompt diagnosis of psoas abscess because of the low sensitivity of imaging tests. Psoas muscle is the main flexor of the hip. This muscle arises from the lateral borders of lower thoracic to lower lumbar vertebrae, passes between the inguinal ligament and the capsule of the hip joint, and ends in the lesser trochanter of the femur. Almost all patients with psoas abscess prefer supine position with moderately flexed knee and mildly externally rotated hip to avoid a strained psoas.^9^ Psoas sign elicits lower abdominal pain by extension or hyperextension of the affected hip and provides a clue to detect regions in the psoas muscle. Its specificity is relatively low because inflammation of adjacent structures such as appendicitis may also cause the same findings.^9^ However, psoas test can deliver important information on the site affected even if the imaging tests did not reveal any abnormalities as in this case.

It is no doubt that modern high‐technology has greatly contributed to medical diagnosis and treatment. However, overconfidence of high‐tech test leads to physicians’ “hyposkillia,” or deficiency of clinical skills due to contempt of history‐taking and physical examination.^10^ As mentioned above, the prompt and precise diagnosis of psoas abscess is not made by high‐tech medicine. Instead, it is only accomplished by high‐touch medicine, or “medicine based on a carefully constructed medical history couples with a pertinent physical examination and critical assessment of the information thus obtained.”^10^ This case explicitly indicates that modern physicians should put much value on history‐taking and physical examination again.

## 3 CONCLUSION

Psoas abscess is an infectious condition with a high mortality rate, which frequently requires surgical drainage. Although imaging tests play a key role in the diagnosis, especially at the early stage, these tests have relatively low sensitivities, albeit a prompt identification of the condition must be important. Physical examination is a cost‐effective diagnostic procedure and sometimes sheds light on the hidden diagnosis in a swifter way than high‐tech procedures such as CT or MRI because of its availability and less invasive nature. In diagnostically challenging cases, physicians should be wary of bedside clinical findings, even if subtle ones.

Here are the key points of this article. Firstly, an early‐stage psoas abscess may be undetectable by imaging tests such as contrast‐enhanced CT. Secondly, no tests except for a thorough bedside physical examination may not provide a clue to detect psoas abscess. Lastly, clinicians should be aware of the sensitivity and specificity of the test they order in order to make a reasonable decision and reach a correct diagnosis.

## CONFLICT OF INTEREST

The authors have no conflict of interest to declare.
